# A comprehensive review on schizophrenia: epidemiology, pathogenesis, diagnosis, conventional treatments, and proposed natural compounds used for management

**DOI:** 10.1007/s00210-025-04351-0

**Published:** 2025-06-13

**Authors:** Alaa Anwar, Aya M. Mustafa, Kareem Abdou, Mostafa A. Rabie, Riham A. El-Shiekh, Ahmed M. El-Dessouki

**Affiliations:** 1 Department of Pharmacognosy, Faculty of Pharmacy, Ain-Shams University, Abbassia, 11566 Cairo Egypt; 2 Department of Pharmacology and Toxicology, Faculty of Pharmacy, Egyptian Russian University, Cairo, Egypt; 3 College of Pharmacy, Al-Ain University, Abu Dhabi, UAE; 4 Department of Pharmacology and Toxicology, Faculty of Pharmacy, Cairo University, Cairo, 11562 Egypt; 5 Department of Pharmacognosy, Faculty of Pharmacy, Cairo University, Kasr El-Aini Street, Cairo, 11562 Egypt; 6 Pharmacology and Toxicology Department, Faculty of Pharmacy6 of October City, Ahram Canadian University, Giza, 12566 Egypt

**Keywords:** Schizophrenia, Natural compounds, MGPCR antagonists, Complementary therapy

## Abstract

Schizophrenia (SCZ) is a complex, comprehensible mental condition that creates alienation from reality. SCZ is a mental disease, which is marked by progressive deficits in working memory, attention, and executive functioning. Because the disease’s etiology is unknown, current psychotherapy and pharmacological treatments merely treat symptoms and do not provide a cure. SCZ symptoms include hallucinations, delusions, disorganized behavior, and lack of desire. Long-term use of antipsychotic (antagonists at multiple G-protein-coupled receptors) (mGPCR antagonists) medicines for therapy has negative health consequences and discourages patients from taking regular medication. Ancient herbal therapies are regaining popularity in disease management due to their natural origins, less side effects, and cost-effectiveness. The various types of phytochemicals include alkaloids, glycosides, polyphenols, terpenes and terpenoids, phytosterols, cannabinoids, and carotenoids could be used as mGPCR antagonists. In this review, we documented the possibility of employing natural compounds as an alternative therapy to treat schizophrenia-related symptoms and cognitive impairments.

## Introduction

Schizophrenia is a serious debilitating disease of adults in every society, affecting around 1–1.5 percent of the global population (Howes and Murray 2014). The incidence of schizophrenia is higher among males than females, with a ratio of 1.4 to 1 (McGrath et al. 2008). Despite its long-standing recognition in psychiatric nosology, schizophrenia remains one of the most contested and conceptually complex diagnoses in mental health. Originating from Kraepelin’s classification of dementia praecox (Kraepelin 1893) and later reframed by Bleuler as “schizophrenia,” the diagnosis has undergone significant transformation over the past century (Bleuler 1911). Contemporary approaches have shifted from psychodynamic interpretations to more neurobiological and dimensional perspectives, yet the category continues to provoke critical debate. Concerns persist regarding its scientific validity, given the broad heterogeneity of symptoms, ranging from hallucinations and delusions to cognitive dysfunction and affective blunting, many of which overlap with other psychiatric conditions (Clementz et al. 2020). Current diagnostic frameworks such as the DSM-5 and ICD-11 rely predominantly on symptom clusters rather than objective biomarkers, which raises questions about diagnostic precision, cultural applicability, and reliability across settings (Association 2019). Ongoing research into genetic, neuroimaging, and pathophysiological markers may refine future diagnostic approaches, potentially leading to more biologically grounded subtyping or a shift toward transdiagnostic frameworks (Orsolini et al. 2022).

Schizophrenia is the eighth costly disorder in the world (McGrath et al. 2008). It is a syndrome that includes both positive and negative symptoms, as well as cognitive issues (McCutcheon et al. 2019). Positive symptoms, including hallucinations and delusions, are the most prominent component of this illness. Negative symptoms include an inability to express emotions and indifference. Cognitive impairments occur before the manifestation of psychosis and can act as a better predictor of the disease (Dienel and Lewis 2019). Unlike other degenerative diseases, it begins in early adulthood or late adolescence (an der Heiden and Häfner 2000). Schizophrenia often develops in the second and third decades of life, however it can also affect the elderly (Brown and Susser 2002). It raises the chance of several brain illnesses, including Parkinson’s disease, autism, Alzheimer’s disease, and multiple sclerosis. Schizophrenia results from a complex combination of genetic, dietary, microbial, and environmental variables (Eyles 2021). Several neurotransmitters, including dopamine, gamma aminobutyric acid (GABA), glutamate, serotonin, and noradrenaline, have important roles in the development and progression of schizophrenia. Furthermore, schizophrenia is caused by a combination of neuroinflammation, oxidative stress, cell signaling pathways, and aberrant immune system activation (Prestwood et al. 2021). Typical mGPCR antagonists medications have a higher affinity, stronger binding, and more inhibition of dopamine receptors than atypical mGPCR antagonists (Bahta et al. 2021). However, atypical mGPCR antagonists medications are more effective than traditional mGPCR antagonists because they operate on dopamine, serotonin, and cholinergic receptors. Individual anti-schizophrenic medications have varying efficacy among patients. Atypical mGPCR antagonists are often more effective but have less side effects than traditional mGPCR antagonists. These synthetic medications have a variety of side effects, including hormonal changes, vertigo, tardive dyskinesia, obesity, infertility, neuroleptic malignant syndrome, drowsiness, and agitation. To avoid these drug-related issues, more effective and safer treatments are desperately needed (Prestwood et al. 2021).

Phytochemicals are natural compounds that provide a cost-effective, accessible, and valuable source of pharmaceuticals. Herbal remedies have been effectively used throughout human history. Humanity is turning to herbal remedies due to the uncertain efficacy and harmful health consequences of previously utilized medication for schizophrenia (Datta et al. 2021). Furthermore, progress in generating synthetic anti-schizophrenic medications remains glacial due to a variety of reasons such as the heterogeneity of schizophrenia phenotypes, confusing pathophysiology, pathological lesions, complex genetic modifications, and other risk factors. As a result of their vast range of biological actions, phytochemicals provide prospective and diversified alternatives to allopathic anti-schizophrenic medications (Yadav 2021). Schizophrenia is typically treated with traditional and atypical mGPCR antagonists, although these medications provide very modest benefits and have a wide range of side effects. Phytochemicals are a broad group of compounds that can be used as an alternative to standard allopathic treatments (Saleem and Akhtar 2022). This review aims to provide a comprehensive overview of schizophrenia, focusing on therapeutic targets and the mechanisms of natural compounds in its management. It also examines experimental models used to study schizophrenia and summarizes current conventional treatments, and clinical trials evaluating the efficacy of these natural treatments.

## Overview of schizophrenia’s disease

### Epidemiology

Schizophrenia is a psychiatric disorder marked by delusions, disorganized speech, hallucinations, and compromised executive functioning. Impacting roughly 1% of the worldwide population, the condition is listed among the top 10 contributors to global disability. The extent to which schizophrenia impairs an individual's daily functioning varies significantly, with some individuals functioning at a high level while others experience severe disability (Srivastav et al. 2022).

In the United States, the average potential life lost for individuals with the illness is 28.5 years. Research findings reveal that schizophrenia psychosis predominantly develops throughout the second and third decades of life, specifically in late adolescence and early adulthood. Schizophrenia manifests first not with psychosis, but with significant impairments in social and cognitive functioning (Velligan and Rao 2023). The male-to-female ratio of schizophrenia burden has remained consistent in the general population over the past 30 years, although it declines from younger to older age cohorts, with raw prevalence in females surpassing that of males after age 65, males exhibiting an earlier age of onset, and females demonstrating a longer life expectancy (Solmi et al. 2023).

It is widely accepted that the prevalence rates for Schizophrenia remain rather consistent globally. Individuals afflicted with schizophrenia frequently have social dysfunction. They are more prone to unemployment, poverty, and homelessness. Regrettably, this results in a reduced life expectancy, with some estimates indicating a drop of 10 to 12 years compared to individuals without schizophrenia. Other studies indicate that, on average, individuals diagnosed with schizophrenia have a life expectancy of approximately 36 years post-diagnosis, and this figure is declining (Volkan 2020).

### Clinical presentation

Schizophrenia is the predominant functional psychotic illness, characterized by diverse symptoms in affected individuals. Contrary to media representations, schizophrenia does not include a “split personality.” It is a chronic psychotic disease that impairs the patient’s cognition and emotional state. The condition frequently disrupts a patient's capacity to engage in social activities and cultivate significant relationships (DiPiro et al. 2014).

**Fig. 1:**
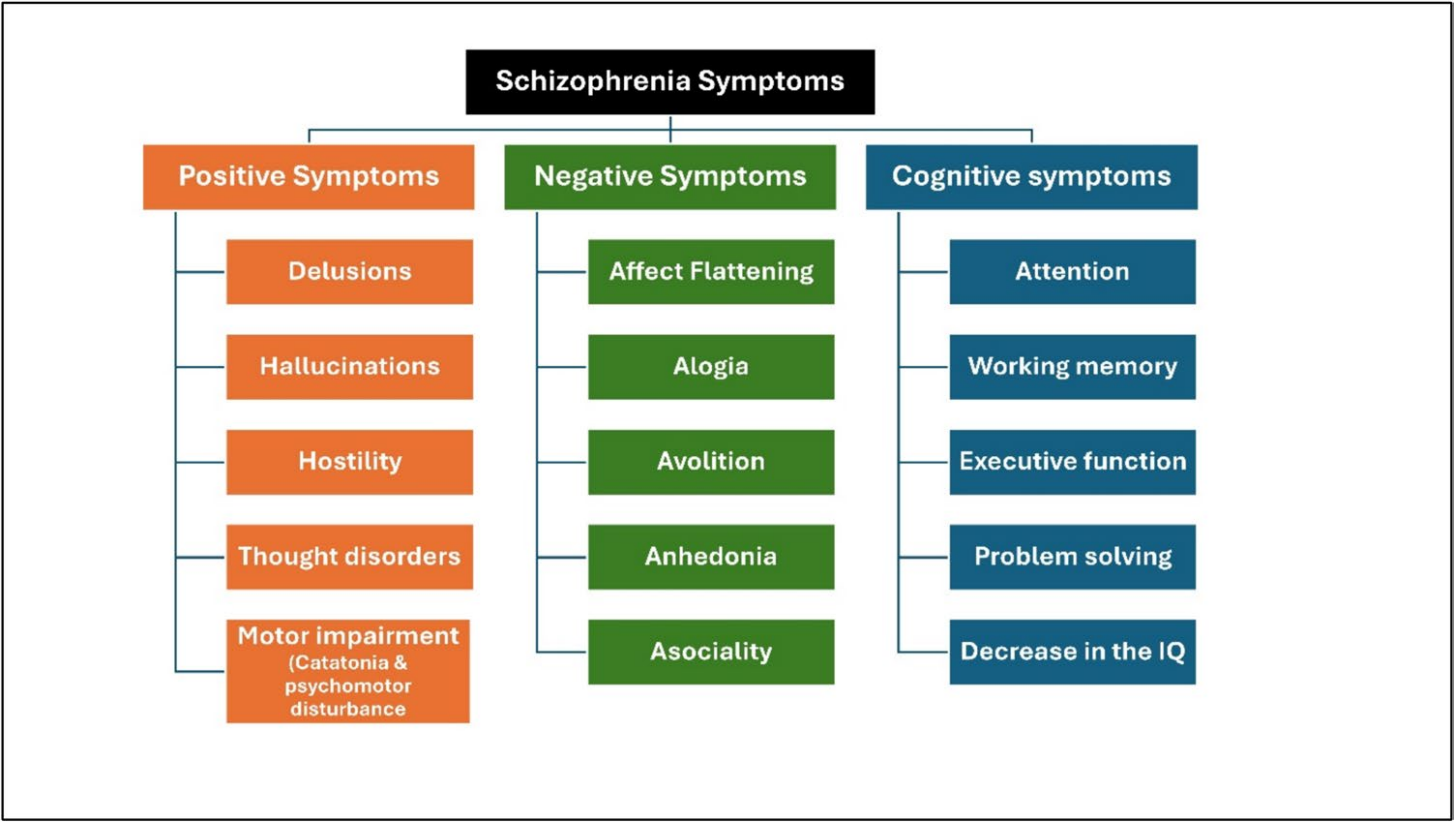
Schizophrenia symptoms

The manifestations of schizophrenia are classified as positive, negative, and cognitive (Fig. 1). Every symptom is crucial as the clinician endeavors to differentiate schizophrenia from other psychotic diseases, including schizoaffective disorder, depressive disorder with psychotic elements, and bipolar disorder with psychotic aspects.

- Positive symptoms: the most readily discernible and reflect an excess or distortion of normal function (delusions and hallucinations) and abnormal motor movement including catatonia and psychomotor disturbance (Habtewold et al. 2020).
- Negative signs: more challenging to identify and linked to significant morbidity due to their impact on the patient’s emotions and behavior. It refers to a diminution or absence of normal behaviors related to motivation and interest (emotional blunting and changes in volition).
- Cognitive changes: These symptoms are generic; hence, they must be sufficiently pronounced for another person to observe them (thought disorder, negativism, autism, and intrapsychic ataxia).

The primary symptoms and comorbidities linked to schizophrenia might result in social and vocational impairment. Functional repercussions encompass insufficient or incomplete education, potentially impairing the patient's capacity to secure and maintain steady employment. Individuals with schizophrenia generally maintain limited social connections and require daily assistance to manage relapses and persistent symptoms (Patel et al. 2014).

### Schizophrenia subtypes

- Paranoid: The occurrence of deceit or hallucinations is prevalent.
- Hebephrenic: A rejection of emotional cognition and an absence of goal-directed behaviour.
- Catatonic: Sustained manifestations of catatonic behaviour, characterized by tremors, agitation, rigidity, and immobility, lasting for a minimum of two weeks.
- Simple: Personal motivation diminishes, and negative consequences become increasingly pronounced (Patel et al. 2014).

### Pathogenesis

Various ideas, including neurodevelopmental and neurochemical theories, have been offered to elucidate the neuropathology of schizophrenia (Birnbaum and Weinberger 2017). Postmortem investigations of the macroscopic and histological pathology of schizophrenia brain tissue revealed reduced brain weight, enlarged ventricular capacity, and atypical neuronal distribution in the prefrontal cortex and hippocampus. Neuro-pharmacological research has validated the participation of dopaminergic, glutamatergic, and GABAergic activity in schizophrenia (Zamanpoor 2020).

**Fig. 2:**
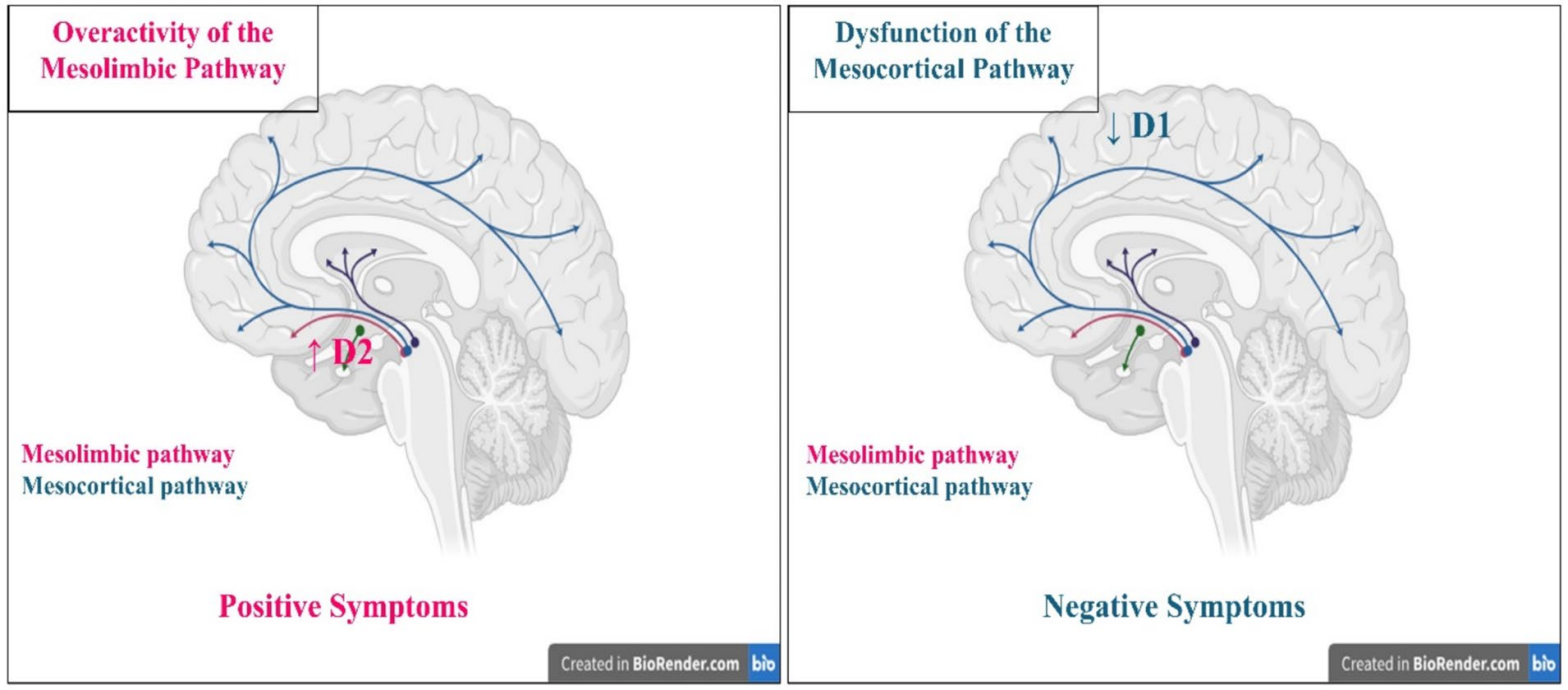
Dopamine hypothesis of schizophrenia pathogenesis (created in https://BioRender.com)

Neurochemical dysfunction of multiple neurotransmitter systems has been suggested to elucidate schizophrenia. The dopamine theory was initially proposed. It has been proposed that psychotic symptoms arise from the activation of dopamine D2 receptors or an excess of dopamine in synapses within mesolimbic dopaminergic pathways. Negative symptoms in schizophrenia are posited to be associated with the hypoactivity of dopamine D1 receptors in the prefrontal brain (Sonnenschein et al. 2020) (Fig. 2).

The glutamate hypothesis posits that GABAergic pathways regulate dopaminergic pathways emanating from the ventral tegmental region. The GABAergic route is regulated by glutamate; in cases of glutamate deprivation, the GABAergic system is unable to be activated and thus cannot regulate the dopaminergic system. Theories about GABAergic system dysfunction propose that the depletion of cortical GABAergic neurons impairs information processing and top-down regulatory mechanisms (Jahangir et al. 2021).

Regarding acetylcholine are predominantly linked to the functioning of nicotinic alpha7 receptors in the hippocampus, and it has been asserted that certain symptoms of schizophrenia stem from dysfunction of this receptor. Besides alpha receptors, muscarinic acetylcholine receptors, particularly M1, M4, and M5, are also implicated in the emergence of psychosis. Moreover, certain experts propose that norepinephrine plays a crucial role in the onset of psychosis and the decline of cognitive skills due to HPA axis malfunction (Kuşman 2024).

### Etiology

Researchers globally have long been engaged with the genesis of schizophrenia. Numerous ideas on the genesis of this disease have emerged, with several theories dominating at different historical intervals. It is noteworthy that scientists revisited certain beliefs after several decades (Oskolkova 2022).

### Genetic

Identifying genes is essential for comprehending the genetic foundation of schizophrenia; nevertheless, the disorder’s variety complicates the identification of its precise genetic base. The genetic foundation of the disease is characterized by an aggregation of risk genes with minimal impact. The identification of susceptibility genes has been facilitated by human genome sequencing and novel DNA amplification techniques. The most often examined variants are single nucleotide polymorphisms (SNPs) and copy number variations (CNVs) involving double nucleotide deletions (Nasrallah 2021).

A recent systematic review indicates that copy number variant (CNV)-based studies have identified five CNV regions associated with schizophrenia, which contain genes that exhibit differential expression in the disorder: PPP1R2 in 3q29, HSPB1 in 7q11.23, INO80E and YPEL3 in 16p11.2, DHRS11 in 17q12, and SEPT5, RTN4R, and SLC2 A11 in 22q11.2 (Merikangas et al. 2022). The CNVs in these regions are linked to neurodevelopmental delays, intellectual disabilities, and various neuropsychiatric phenotypes, including anxiety (3q29, 7q11.23, and 17q12), autism spectrum disorder (ASD; 3q29, 7q11.23, 16p11.2, 17q12, and 22q), attention-deficit/hyperactivity disorder (ADHD; 7q11.23 and 22q), and bipolar disorder (3q29, 7q11.23, and 17q12), as well as immune system dysfunction, cardiac pathologies, and numerous other medical conditions (Hubbard et al. 2021). The most extensively studied copy number variation (CNV) linked to an elevated risk of schizophrenia is the 22q11.2 deletion syndrome, which correlates with a 25-fold increase in the chance of developing schizophrenia (Cleynen et al. 2021).

Three polymorphisms of the PRODH gene are linked to an elevated risk of schizophrenia. Infrequent gene variations have demonstrated a reduction in enzyme activity, resulting in atypical plasticity of glutamatergic synapses and dysregulation of dopamine in the frontal brain. The COMT gene, situated in the 22q11 region, has been examined for its function in dopamine degradation and its polymorphisms. The high activity allele seems to elevate the risk of schizophrenia and impair executive function, a domain disrupted in the disorder (Shen et al. 2023). Dystrobrevin-binding protein 1 (DTNBP1), also known as dysbindin, has been found using fine-mapping of the 6p24-22 locus (Belužić 2021).

The Neurolregulin 1 (NRG1) gene, the disrupted schizophrenia 1 (DISC1) gene, and the trace amine receptor 4 (TAAR6) gene have been meticulously mapped and recognized as potential susceptibility genes for schizophrenia. The NRG1 gene exhibits variation in its linkage disequilibrium structure and the presence of many risk alleles. The DSC1 gene is associated with schizophrenia owing to allelic heterogeneity, and its complexity is corroborated by polymorphisms that affect hippocampus anatomy and function. The trace amine receptor 4 (TAAR6) gene has been precisely mapped and recognized as a positional possibility for schizophrenia (Walgama 2024). The 13q32-34 locus has been precisely delineated, and G72 regulates D-amino acid oxidase activity, influencing glutamatergic signaling. The Epsin 4 gene has been precisely mapped, revealing four haplotypes that exhibit linkage disequilibrium with schizophrenia. A distinct meta-analysis discovered additional related genes throughout the dopaminergic and serotonergic systems, along with those influencing neurodevelopment (Jawanjal and Chatterjee 2021).

### Environmental factors

Environmental factors correlate with cognitive capacities, particularly in contexts of socioeconomic deprivation. The heightened occurrence of adverse environmental influences in individuals with schizophrenia suggests that analogous mechanisms may apply to cognitive deficits in schizophrenia as they do in the general population (Orsolini et al. 2022).

Acute cannabis consumption is linked to significant cognitive impairment, and habitual users tend to exhibit inferior performance on cognitive assessments. Nevertheless, among individuals with schizophrenia, certain research indicate that cannabis users exhibit superior cognitive function compared to non-users. This conclusion seems paradoxical, considering cannabis consumption in healthy participants and other schizophrenia studies has been linked to cognitive function abnormalities (Hjorthøj et al. 2023).

Residing in an urban setting is associated with an increased prevalence of schizophrenia, however the causal relationship remains ambiguous. This connection may be influenced by elevated degrees of socioeconomic deprivation. In the overall population, socioeconomic disadvantage correlates with inferior educational achievement, likely due to the association of rich neighborhoods with enhanced cognitive stimuli. Nonetheless, there seem to be supplementary elements connecting cognition and urbanicity (Abrahamyan Empson et al. 2020).

In preterm children, residing in an urban environment correlates with decreased cognitive development scores, even after accounting for socioeconomic considerations. In the general populace, residing in an urban setting correlates with diminished spatial navigation skills, whereas air pollution is linked to impaired cognitive function and a heightened risk of schizophrenia development. Childhood trauma exposure correlates with a heightened risk of schizophrenia and diminished cognitive performance during childhood and adolescence (Budisteanu et al. 2020).

Individuals with schizophrenia exhibit an additional deterioration in cognitive function in later life. The latter may indicate a heightened prevalence of smoking, obesity, and hyperglycemia, which can negatively impact cerebrovascular function. Hypertension, diabetes, and metabolic syndrome are all correlated with markedly impaired cognitive functioning in persons with schizophrenia. Other variables encompass the deficiency of social and vocational stimulation linked to the illness (McCutcheon et al. 2023).

### Neuro-inflammation

Inflammation is a biological process that mobilizes the immune system to protect against threats, reducing tissue damage and averting systemic dissemination. Schizophrenia is associated with persistent, mild activation of inflammation and the immune system. The assessment of inflammation and immunological activation necessitates the utilization of indicators like cytokines, which have been investigated as potential biomarkers for schizophrenia. Elevated serum interleukin-6 (IL-6) and tumor necrosis factor-α (TNF-α) are observed during acute psychotic relapses in individuals with schizophrenia, and IL-6 levels decrease following mGPCR antagonists treatment of the acute episode (Mongan et al. 2020).

Meta-analyses have shown elevated pro-inflammatory cytokines in individuals with diagnosed schizophrenia, medication-naïve patients experiencing their first episode of psychosis, and those at clinical high risk for psychosis. Nevertheless, research assessing inflammatory biomarkers in psychosis have frequently employed cross-sectional designs, making it impossible to clearly infer the temporal link between exposure (inflammation) and result (psychosis) (Misiak et al. 2021). Longitudinal studies have sought to mitigate this constraint, indicating that the rise in inflammatory tone transpires at an early stage, prior to the emergence of overt psychotic symptoms. Biomarker studies contribute to the evidence of inflammation and immunological dysregulation in psychosis and may possess translational utility for diagnostic or prognostic significance (Morrens et al. 2022).

Besides their correlation with illness, inflammatory cytokines may function as indicators or predictors of therapeutic efficacy. Treatment-resistant patients exhibit increased levels of TNF receptor 1, IL-2, IL-6, IL-8, and IL-10. In patients with first-episode psychosis, elevated plasma concentrations of IL-6 and IFN-γ were observed at baseline and after 12 weeks of standard mGPCR antagonists therapy in non-responders relative to responders (Dunleavy et al. 2022).

### Diagnosis

Schizophrenia is characterized as a diverse clinical illness that exhibits similarities with various other psychological disorders, complicating its diagnosis. Schizophrenia is presently classified as an illness with subgroups to account for its heterogeneity. Nevertheless, subclasses rely on certain shared clinical characteristics that render the diagnosis ambiguous. The diagnosis of schizophrenia is determined according to the Diagnostic and Statistical Manual of Mental Disorders. Patients must exhibit two or more of the specified symptoms for a duration of 1 month. The symptoms encompass delusions, hallucinations, incoherent speech, severely disorganized or catatonic behavior, and negative symptoms (including emotional flatness, alogia, and avolition) (Earl 2017).

The start of schizophrenia varies among individuals, ranging from abrupt onset to a prolonged prodromal phase. Negative symptoms may be seen around 5 years prior to the onset of the initial psychotic episode. Recent advancements in the identification of schizophrenia-related loci, along with the delineation of specific schizophrenic symptoms, suggest that the linked SNPs may serve as possible biomarkers to aid in the diagnosis of schizophrenia in the genomic era (Zamanpoor 2020).

### Therapeutic targets/mechanisms of actions of natural compounds in the management of Schizophrenia

Schizophrenia is a complex psychiatric disorder that is characterized with positive, negative, and cognitive symptoms. Conventional therapeutic approaches target glutamatergic, dopaminergic and cholinergic pathways with limited efficacy for negative and cognitive symptoms (Hardingham and Do 2016). The lack in efficacy, unnecessary side effects and resistance oblige to discover novel therapeutic approaches. The development of novel schizophrenia therapies necessitates deep understanding of disease etiology (Lewis and Hashimoto 2007; Drozdz et al. 2023; Fond et al. 2023).

### Targeting the glutamatergic pathway

One of the pathological hubs of schizophrenia is the N-methyl-D-aspartate (NMDA) receptor hypofunction (Coyle 2012) which affects the function of fast-spiking parvalbumin-positive interneurons (PVIs) in the prefrontal cortex (PFC) (Hardingham and Do 2016). Previous human studies confirmed that NMDAR hypofunction might be a cause for schizophrenia by observing schizophrenia-like symptoms after administering NMDAR antagonists(Hardingham and Do 2016). A genetic study that utilized exome sequencing has indicated schizophrenia-linked mutations in NMDAR subunit genes GRIN2 A and GRIN2B (Tarabeux et al. 2011). Moreover, several studies suggested that NMDAR blockade may decrease PVIs number which causes cortical disinhibition, hence altering excitation-inhibition (E/I) balance (Wang et al. 2013; Hardingham and Do 2016). This imbalance was sufficient to induce schizophrenia-like phenotypes (Lewis et al. 2012). Furthermore, there is growing evidence that deficits in cortical neurons expressing gamma-amino butyric acid (GABA) is a hallmark of schizophrenia (Lewis et al. 2005, 2012). Reduced glutamic acid decarboxylase (GAD67), an enzyme involved in GABA synthesis, expression has been observed in several postmortem studies (Lewis et al. 2005).

A previous study used quercetin, a natural flavonoid that acts as a negative allosteric modulator for GABA_A_ receptors, to restore the glutamatergic transmission in the PFC and, successfully reducing the positive symptom in mice (Schwartz 2016; Fan et al. 2018). Mechanistically, quercetin could inhibit GABA_A_ receptors on defective PVIs, hence enhancing their activation and subsequently reinstating adequate levels of inhibition onto prefrontal pyramidal neurons (Schwartz 2016; Fan et al. 2018). This hypothesis is supported by the fact that PVIs are more abundant than excitatory neurons in the PFC.

### Targeting the redox balance

Redox imbalance is highly linked to schizophrenia since it increases the oxidative stress which leads to macromolecular damage in CNS and schizophrenia-like behavior (Dringen 2005; Dringen et al. 2005). Several studies on patients with schizophrenia has demonstrated increased protein oxidation and decreased level of vitamin C and reduced glutathione (GSH) (Flatow et al. 2013). The GSH system is highly dysregulated in schizophrenia, especially glutathione reductase (GR) and glutathione peroxidase (GPX) (Yao and Keshavan 2011). GPX catalyzes the reduction of cellular peroxides, while GR catalyzes the regeneration of GSH. In rodents, inhibiting the glutamate-cysteine ligase enzyme depleted the brain GSH, inducing cognitive symptoms like that observed in schizophrenia (Gysin et al. 2007; Matsuzawa and Hashimoto 2011). A potential antioxidant therapy for schizophrenia is N-acetylcysteine (NAC) which provides the brain with the required amino acids for GSH generation (Breier et al. 2018; Sepehrmanesh et al. 2018; Tharoor et al. 2018). NAC prevents oxidative stress in a developmental rat model of schizophrenia and inhibits both electrophysiological and behavioral abnormalities (Ghaderi et al. 2019b; Fond et al. 2023). It has been proved that NAC is an effective adjunct for the treatment of negative symptoms in schizophrenia (Tharoor et al. 2018).

### Targeting the cholinergic system

Neuronal nicotinic acetylcholine receptors (nAChRs) are ligand-gated ion channels that exist in both excitatory neurons and GABAergic interneurons. Abnormal expression of α7 nAChR has been linked to abnormalities in the auditory sensory gating which is commonly found in patients with schizophrenia (Young and Geyer 2013; Caton et al. 2020).

Muscarinic receptors are G-protein-coupled receptors extensively allocated in the brain. The M1 receptor is the most abundant muscarinic receptor in the mammalian brain (Eglen 2005). Previous study showed alleviation of negative symptoms in schizophrenia patients by the anticholinergic agent trihexyphenidyl which binds to M1 muscarinic receptor (Giachetti et al. 1986).

Furthermore, there is an established crosstalk between cholinergic interneurons and dopaminergic neurons in the basal ganglia (Kuo and Liu 2019). Activation of excitatory postsynaptic nAChRs induces action potential in dopaminergic neurons in the ventral tegmental area through triggering glutamate release in glutamatergic synapses (Zhao-Shea et al. 2011; Faure et al. 2014). Modulation of dopamine neurotransmission through nicotinic and muscarinic receptors could underlie psychosis and dyskinesia observed in patients with schizophrenia (Bordia et al. 2016).

### Linking multi-drug targets

PVIs are vulnerable to redox imbalance. For example, pharmacological depletion of GSH in brain during development leads to PVI deficits in the medial prefrontal cortex (Wirth et al. 2010). Previous study presented causal evidence for linking oxidative stress with PVI abnormalities through preventing PVI deficits in neonatal ventral hippocampal lesion (NVHL) model upon NAC treatment (Johnson et al. 2013; Cabungcal et al. 2014). PVI defects after redox imbalance could be attributed to the loss of ensheathing perineuronal nets (Hardingham and Do 2016).

Recently, it has been shown that GSH dysregulation can induce NMDAR deficits which are linked to cognitive decline (Steullet et al. 2006; Guidi et al. 2015). Reciprocally, NMDAR hypofunction results in redox imbalance, oxidative stress and GSH deficits (Steullet et al. 2006; Hardingham and Do 2016). Mechanistically, cortical NMDAR malfunction decreases interneurons activity, leading to excitation–inhibition (E/I) imbalance and PVI deficits. These deficits cause cortical disinhibition which increases neuronal interleukin-6 (IL-6) production and oxidative stress through activation of NADPH oxidase, which generates H_2_O_2_ (Wang et al. 2013). Collectively, all these abnormalities lead to altered behavior and sensory processing and cognitive deficits in schizophrenia.

### Neurotrophic factor enhancement

One of the main signaling pathways involved in schizophrenia is brain-derived neurotrophic factor (BDNF) pathway (Ahmad et al. 2023). BDNF promotes the development of GABAergic interneurons, especially PVIs through the interaction with its receptor tyrosine kinase B (TrkB) (Gliwinska et al. 2023; Zhang et al. 2023). Enhanced BDNF expression improves neuroplasticity and neurogenesis. Recent study on schizophrenic patients has demonstrated reduced expression of both BDNF and TrkB which is correlated with reduced expression of GABA-related proteins, including parvalbumin and glutamic acid decarboxylase (GAD67) (Xu et al. 2000a; Xu et al. 2000b; Yamada et al. 2002).

Current therapeutic avenues rely on the use of multi-target compounds, such as curcumin and *Ginkgo biloba* extract (GBE). Curcumin is a natural non-flavonoid polyphenolic compound which has antioxidant and anti-inflammatory effects (Miodownik et al. 2019). It is a lipophilic molecule, so it can pass the blood–brain barrier, exerting its antioxidant and anti-inflammatory actions through reducing the production of reactive oxygen species and inflammatory cytokines, respectively (Kucukgoncu et al. 2019; Moghaddam et al. 2021; Dinakaran et al. 2022). Several studies have demonstrated significant behavioral improvements upon adding curcumin to the treatment regimen of schizophrenic patients (Dinakaran et al. 2022; Bulnes et al. 2023; Rabiee et al. 2023). Moreover, it has been shown that curcumin enhances the expression of BDNF and alleviates positive, negative and cognitive symptoms in preclinical and clinical studies (Wynn et al. 2018).

On the other hand, GBE has been used in several neuropsychological disorders, including schizophrenia due to its antioxidant, anti-inflammatory, and neuroprotective properties (Chen et al. 2012; He et al. 2012; Brondino et al. 2013; Tian et al. 2013). Previous clinical studies demonstrated behavior improvements upon using GBE as adjunctive therapy in patients with schizophrenia (Atmaca et al. 2005; Dinakaran et al. 2022).

### Current treatment options for schizophrenia

**Fig. 3:**
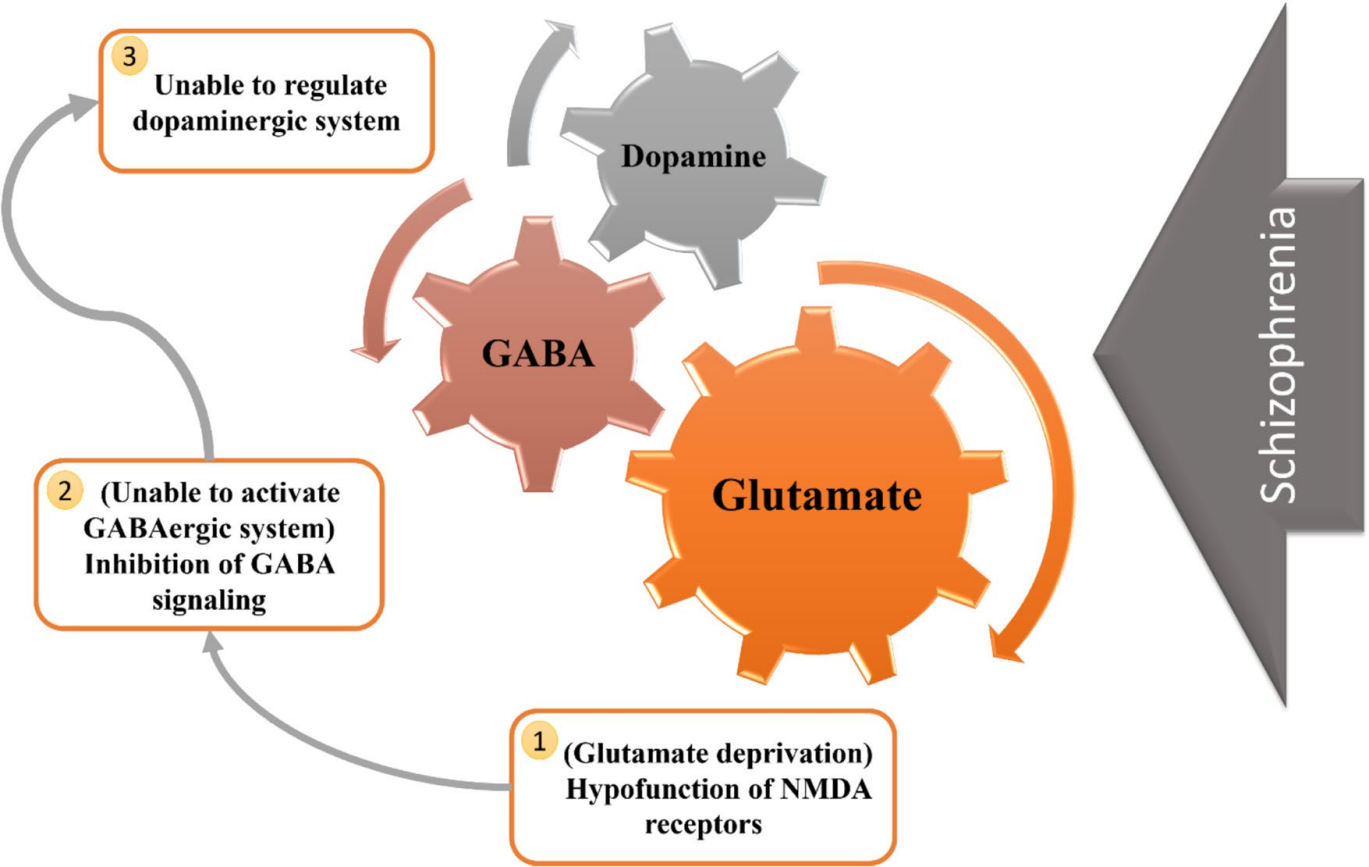
Schematic figure illustrating the GABAergic and glutamatergic mechanisms involved in schizophrenia

Schizophrenia treatment primarily relies on mGPCR antagonists medications (Tables 1 and 2). However, these therapies are often associated with significant side effects and limited efficacy, particularly in addressing cognitive and negative symptoms. Despite advancements, treatment resistance remains a challenge, necessitating the exploration of complementary strategies. The integration of natural compounds into existing pharmacotherapy represents a promising avenue for improving outcomes by mitigating side effects, enhancing therapeutic efficacy, and providing neuroprotective benefits (Hoenders et al. 2018; Hynes et al. 2020; Asgharian et al. 2022) (Fig. 3).

**Table 1:** Comparison of first-generation and second-generation mGPCR antagonists used in schizophrenia treatment

	Typical mGPCR antagonists (First-Generation; FGA)	Atypical mGPCR antagonists (Second-Generation; SGA)
Example	Haloperidol and Chlorpromazine	Clozapine, Olanzapine, and Risperidone
Mechanism of action	Primarily block dopamine D2 receptors, reducing dopaminergic activity (Fig. 3)	Block both dopamine D2 and serotonin 5-HT_2 A_ receptors, leading to a more balanced neurotransmitter modulation (Fig. 3**)**
Efficacy	Effective in reducing positive symptoms (hallucinations, delusions)	More effective for negative symptoms (apathy, social withdrawal) and cognitive impairment (Kadakia et al. 2022; Martinotti et al. 2022); Clozapine is particularly useful for treatment-resistant schizophrenia
Side effects	High risk of extrapyramidal symptoms (EPS), including tremors, rigidity, dystonia, and tardive dyskinesia. Long-term use can cause neuroleptic malignant syndrome (NMS), a severe, life-threatening condition characterized by hyperthermia and autonomic dysfunction (Vaiman et al. 2021; Kim 2022; Wijdicks and Ropper 2024)	Lower risk of EPS but increased metabolic side effects such as weight gain, insulin resistance, and cardiovascular issues (Vazquez-Bourgon et al. 2020). Clozapine requires regular blood monitoring due to the risk of agranulocytosis (Magistri and Mellini 2022)
Other Considerations	Generally, more affordable but associated with poorer tolerability due to motor side effects	Preferred for long-term treatment due to better tolerability but requires monitoring for metabolic and hematologic adverse effects

This table contrasts typical (first-generation) and atypical (second-generation) metabotropic G protein-coupled receptor (mGPCR) antagonists commonly used in the management of schizophrenia. While FGAs primarily target dopaminergic pathways and are effective against positive symptoms, they are associated with significant motor side effects. SGAs, on the other hand, target both dopaminergic and serotonergic receptors, offering improved efficacy for negative and cognitive symptoms with a better side effect profile, albeit with metabolic risks

**Table 2:** Emerging therapeutic strategies targeting non-dopaminergic pathways in schizophrenia

Therapeutic Approach	Mechanism of Action	Potential Benefits
Glutamate-Modulating Agents	Enhance NMDA receptor function using compounds like glycine, D-serine, and sarcosine	May improve cognitive deficits and negative symptoms by addressing NMDA receptor hypofunction (Pei et al. 2021)
Anti-Inflammatory and Antioxidant Agents	Reduce oxidative stress and neuroinflammation through omega-3 fatty acids, N-acetylcysteine (NAC), and flavonoids	Could provide neuroprotection, improve mitochondrial function, and enhance cognitive and functional outcomes (Castro Zamparella et al. 2022; Schoeps et al. 2022)
Hormonal and Endocrine-Based Therapies	Address hormonal dysregulation using selective estrogen receptor modulators (SERMs) and thyroid hormone supplementation	May improve treatment outcomes, particularly in female patients, by modulating estrogen and thyroid hormone levels (Rasool et al. 2021; Mu et al. 2024)

This table summarizes novel non-dopaminergic therapeutic approaches under investigation for the treatment of schizophrenia. These include agents targeting glutamatergic neurotransmission, inflammation and oxidative stress, and hormonal dysregulation. By expanding treatment strategies beyond dopamine receptor antagonism, these emerging therapies aim to address cognitive deficits, negative symptoms, and improve overall functional outcomes in patients with schizophrenia

## Experimental models for studying schizophrenia and natural compounds

**Fig. 4:**
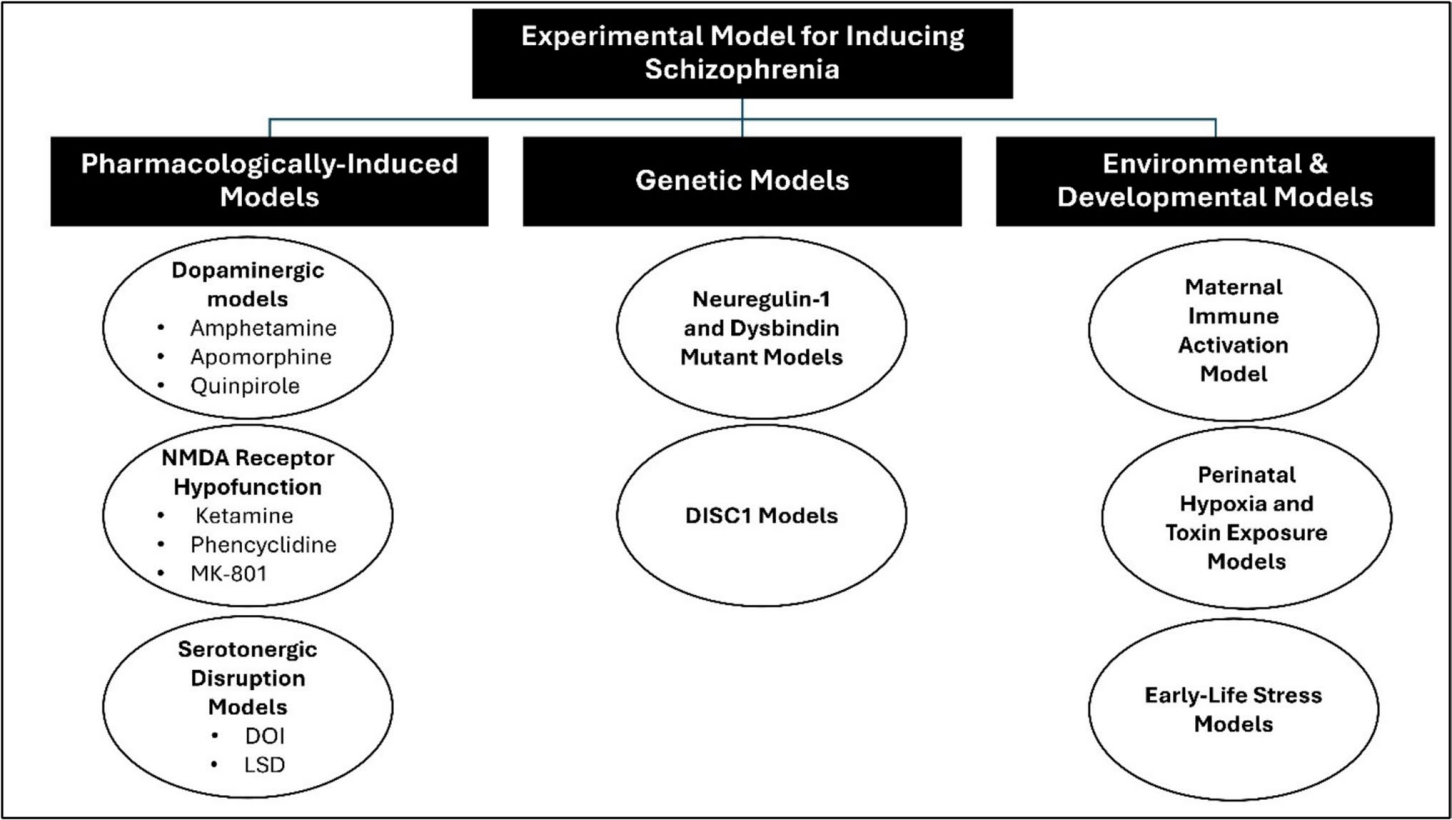
Experimental models for schizophrenia induction

These experimental models broadly encompass genetic, pharmacological, neurodevelopmental, and environmental approaches, each recapitulating distinct features of schizophrenia (Fig. 4). With its multifaceted etiologies, schizophrenia necessitates robust experimental models to investigate its pathophysiology and potential therapeutic interventions, including natural compounds (Białoń and Wąsik 2022; Krzyściak et al. 2024). Understanding these models allows researchers to simulate various pathophysiological mechanisms underlying the disorder and test novel therapeutic strategies, including naturally derived compounds that may exhibit neuroprotective, anti-inflammatory, and antioxidant properties (Marino et al. 2022; Saleem and Akhtar 2022; Pekdemir et al. 2024).

## Pharmacologically induced models

Pharmacological models used agents that disrupt neurotransmission, mimicking schizophrenia-like symptoms (Table 3). These models serve as critical tools for assessing both conventional antipsychotic drugs and alternative therapeutic agents such as natural compounds, which may offer neuroprotection with fewer side effects compared to synthetic drugs (Asgharian et al. 2022).

**Table 3:** Preclinical pharmacological models of schizophrenia and associated natural compounds

Model Type	Agents Used	Mechanism of Action	Symptoms Mimicked	Applications in Schizophrenia Research	Potential Natural Compounds Tested
**Dopaminergic Models** (McCutcheon et al. 2020; Morales-Medina et al. 2021; Alsharif et al. 2023) (Molitch 2020; Vartzoka et al. 2023; Dorogan et al. 2024)	Amphetamine, Apomorphine, Quinpirole	Excessive dopamine release or direct stimulation of dopamine receptors, particularly in the mesolimbic pathway	Positive symptoms: hallucinations, delusions, hyperlocomotion, stereotyped behavior	Evaluates dopamine-related pathophysiology and the efficacy of dopamine-modulating treatments	Flavonoids, Alkaloids
**NMDA Receptor Hypofunction Models** (Güneri et al. 2021; Brakatselos et al. 2024; Li et al. 2024a) (Raju et al. 2023)	Ketamine, Phencyclidine (PCP), MK-801	Inhibition of NMDA receptors, leading to disrupted glutamatergic neurotransmission and synaptic dysfunction	Cognitive deficits, working memory impairment, social withdrawal, negative symptoms	Models schizophrenia-related cognitive dysfunction and evaluates NMDA-enhancing or neuroprotective treatments	Polyphenols, Flavonoids, Alkaloids
**Serotonergic Disruption Models** (Hermle and Kraehenmann 2018; Desouza et al. 2021; Fiorentini et al. 2021; Nikolaus et al. 2024)	2,5-dimethoxy-4-iodoamphetamine (DOI), Lysergic Acid Diethylamide (LSD), 5-HT Agonists/Antagonists	Dysregulation of serotonin receptors, particularly 5-HT2 A, affecting cognition and mood	Cognitive deficits, emotional dysregulation, negative symptoms, hallucinations	Investigates the role of serotonin in schizophrenia and the potential of serotonergic modulators	Curcumin, Resveratrol

This table summarizes the main pharmacological animal models used in schizophrenia research, categorized by neurotransmitter system dysregulation. Each model is characterized by specific agents, mechanisms of action, and the behavioral or cognitive symptoms it mimics. These models are essential for understanding the neurobiology of schizophrenia and for preclinical screening of both synthetic and natural therapeutic candidates

## Genetic models

Genetic animal models involve gene knockouts or mutations that resemble schizophrenia-related abnormalities. These models provide insight into genetic susceptibility factors and allow the investigation of natural compounds that target oxidative stress, mitochondrial dysfunction, and neuroinflammation, key pathological processes in schizophrenia (Beeraka et al. 2022).

- Neuregulin-1 (NRG1) and dysbindin mutant models: These models, involved in synaptic plasticity and glutamatergic signaling, facilitate the evaluation of neuroprotective phytochemicals. Neuregulin-1 plays a crucial role in synaptic maturation and neural development, while dysbindin is implicated in synaptic vesicle function (Năstase et al. 2022; Rodríguez Prieto 2024). Phytochemicals such as quercetin and epigallocatechin gallate (EGCG) have been tested for their ability to modulate synaptic function and protect against oxidative damage (Uddin et al. 2020).
- DISC1 (disrupted in schizophrenia 1) models: DISC1 is a key neurodevelopmental protein linked to structural and functional abnormalities in schizophrenia. Deficiencies in DISC1 lead to disrupted neuronal migration, impaired synaptic plasticity, and cognitive dysfunction (Sasani and Erbaş, 2022; Mısır and Akay 2023). Investigating natural compounds such as ginseng and omega-3 fatty acids in these models helps identify potential interventions that promote neurogenesis and synaptic repair (Park et al. 2020; Sasikumar et al. 2022).

## Environmental and developmental models

Prenatal and postnatal environmental insults contribute to schizophrenia pathogenesis. These models simulate risk factors such as maternal infections, hypoxia, and exposure to environmental toxins. They are used to assess natural compounds with antioxidant, anti-inflammatory, and neuroprotective effects (Lipner et al. 2022; Kawikova et al. 2024).

- Maternal immune activation (MIA) model: Induced by infections or inflammatory agents (e.g., poly(I:C)), this model is used to evaluate anti-inflammatory and neuroprotective natural compounds such as curcumin, resveratrol, and flavonoids. Chronic neuroinflammation is a major contributor to schizophrenia, and these compounds have been explored for their ability to reduce inflammatory cytokine expression and restore normal neurodevelopmental trajectories (Haddad et al. 2020; Zhong et al. 2020; Choudhury and Lennox 2021).
- Perinatal hypoxia and toxin exposure models: These models induce oxidative stress and neurodevelopmental impairments, providing a testing platform for antioxidant-rich phytochemicals. Hypoxic conditions during prenatal development are associated with increased risk of schizophrenia, and natural compounds such as sulforaphane and green tea polyphenols have shown promise in counteracting oxidative stress and neuronal damage (Nalivaeva et al. 2018; Curpan et al. 2021; Gummerson 2023).
- Early-life stress models: Psychological and social stressors in early life are significant risk factors for schizophrenia. Rodent models subjected to maternal separation or chronic mild stress exhibit schizophrenia-like symptoms, including cognitive deficits and social withdrawal (Vafadari et al. 2019; Cavichioli et al. 2023). Adaptogenic herbs such as *Rhodiola rosea* and *Withania somnifera* (Ashwagandha) have been evaluated for their ability to modulate stress responses and support cognitive resilience (Speers et al. 2021; Ivanova Stojcheva and Quintela 2022).

## Natural compounds interacting with one or more targets and evidence supporting their use in the management of Schizophrenia

Synthetic approved anti-schizophrenic drugs may cause several problems. Typical antipsychotic medications only affect positive symptoms and can cause extra-pyramidal side effects such as dyskinesia and akathisia. Atypical medications have different risks and challenges where they can increase the risk of cardiovascular diseases, diabetes, and agranulocytosis. Additionally long-term usage of atypical mGPCR antagonists can lead to uncontrollable movements, such as tics and tremors, excessive sleep, dizziness, convulsions, constipation, and nausea (Amato et al. 2017).

Researchers have been continually exploring the natural realm for alternative sources of drug leads that demonstrate significant bioactivity. Natural compounds are being intensively searched for due to numerous reasons such as their accessibility, availability, affordability, efficacy, biocompatibility, perceived safety, and eco-friendliness. Moreover, medicinal plants offer a high structural and functional diversity of bioactive compounds, which are difficult to synthesize artificially (Odebunmi et al. 2022).

Several efforts have been made by scientists over the years to study and examine the antipsychotic potential of natural compounds. Many classes of natural products belonging to alkaloids, xanthones, terpenoids, anthraquinones, polyphenolics, flavonoids, and others are considered as potential drug leads for the management of schizophrenia. This is demonstrated by several mechanisms of action by the wide chemical variety of these compounds. Secondary metabolites exert their effects by agonist/antagonist actions on psychosis-related neurotransmitters receptors, antioxidant, anti-inflammatory activities, direct effects on enzymes such as prolyl oligopeptidase, and poly (ADP-ribose) polymerase (PARP) enzymes, and many compounds were capable of modulation of multiple signaling pathways simultaneously (Küpeli Akkol et al. 2021; Asgharian et al. 2022).

Natural products highlighted in this section were used either alone or as an adjuvant therapy with conventional drugs owing to their antipsychotic potentials dealing with one or more of the disease targets. This resulted in synergistic effects, leading to the reduction of the dosing of the chemical drug, which eventually will reduce the reported side effects, and lead to better management of the disease and higher patient compliance rates. For example, Berberine was used in clinical study as an add-on therapy with atypical antipsychotic drug, improving negative symptoms (Li et al. 2022). Another study explained that berberine may alleviate the metabolic side effects of the antipsychotic drugs (Shi et al. 2025). Another case clearly demonstrating the positive influence of natural products as adjuvant therapy was in a clinical case study when quercetin was added to the treatment plan of a patient who has been receiving antipsychotic drugs with no significant improvement in symptoms. Positive and negative symptoms were improved after 2 months of the addition. A second case was reported where significant improvements were achieved including irritability reduction, both verbal and behavioral disorganization and thought disorders amelioration. This was achieved when quercetin was added with clozapine in the treatment regimen (Schwartz 2016). Table 4 demonstrates diverse compounds that have shown remarkable activity against schizophrenia either in vitro, in vivo, and/or in clinical trials.

**Table 4:** Natural products and their evidence-based mechanism of action in managing Schizophrenia

Class of Compounds	Compound name	Reported physiological activity against SCN	Reference(s)
**Alkaloids**			
Indole alkaloids	Alstonine	Increasing serotonergic transmission Reducing glutamate uptake by 5-HT_2 A_ and 5-HT_2 C_ serotonin receptors Modulating dopamine (DA) uptake Increasing intracellular glutathione levels	(Olawale et al. 2023)
Isoquinoline alkaloids	(‒)-Stepholidine	Dopamine (DA) D1 receptor partial agonist and D2 antagonist in the nigrostriatal and mesocorticolimbic dopaminergic pathways, thus improving both the negative and positive symptoms of SCZ	(Yang et al. 2007)
	Berberine	Inhibition of prolyl oligopeptidase (POP) enzyme with IC_50_ of 145 ± 19 μM Adjunctive therapy with atypical antipsychotic drugs improves negative symptoms through anti-inflammatory effects Alleviation of antipsychotic-associated weight gain and metabolic syndrome	(Tarrago et al. 2007; Li et al. 2022; Shi et al. 2025)
Benzazepine alkaloids	Galanthamine	Acetylcholinesterase competitive inhibitor Type I positive allosteric modulator of *α*7nACh receptors Alleviates cognitive impairments	(Koola et al. 2020)
Pyridine alkaloids	Arecoline	Partial agonist of the acetylcholine muscarinic receptor Acts by directly targeting oligodendrocytes, and inhibiting demyelination of white matter It increases social and cognitive functions	(Hussain et al. 2018)
Sesquiterpene alkaloids	Huperzine A	Reversible inhibitor of acetylcholinesterase (AChE)	(Wang et al. 2006)
**Xanthones**	Mangiferin	Ameliorates Memory impairment associated with SCN Improves spatial recognition, episodic aversive events, short- and long-term memories owing to its antioxidant and anti-inflammatory activities	(Lum et al. 2021)
**Terpenoids**			
Diterpenoids	Incensole	Competitive inhibitors of POP enzyme with IC_50_ values ranging from 3.1 ± 0.45 to 24.4 ± 1.16 μM SAR: The activity may be contributed to the 14-membered ring cycle of the diterpene based compounds	(Khan et al. 2022)
	Incensole acetate		
	Incensone		
	Incensfuran		
	*epi*-Incensole acetate		
Triterpenoids	Galphimine-A	They displayed interaction with the dopaminergic and glutamatergic systems, modifying the behavioral and psychotic symptoms in ketamine-induced mice models	(Santillán-Urquiza et al. 2018)
	Galphimine-B		
	Galphimine-C		
	Maslinic acid	Single dose of 30 mg/kg blocked the MK-801-induced hyperlocomotion and reversed the MK-801-induced sensorimotor gating deficit It alleviated the social behavior deficits, reversed attention and recognition memory impairment It normalized the phosphorylation levels of Akt-GSK-3b and ERK-CREB in the prefrontal cortex	(Jeon et al. 2017)
	Oleanolic acid	Ameliorating MK-801-induced psychosis-like symptoms, such as spontaneous hyperlocomotion, PPI deficits of sensorimotor gating disruption, and object recognition impairments in mice	(Park et al. 2014)
Triterpenoid saponins	Polygalasaponins	Displayed dose-related reduction in the apomorphine-induced climbing behaviour, the 5-HTP-induced serotonin syndrome, the MK-801-induced hyperactivity and the cocaine-induced hyperactivity in mice models. Hence, they have both dopamine and serotonin receptor antagonist properties	(Chung et al. 2002)
Triterpene glycosides	Mogroside V (Mog V)	Mog V: -Prevention of Prepulse inhibition (PPI) deficits and social withdrawal induced by MK-801 -Alleviation of cellular and neurochemical responses of MK-801 in medial prefrontal cortical cortex (mPFC) Mog V and 11-oxo-Mogrol: -Amelioration of neuronal damage by Promoting neurite outgrowth, inhibiting cell apoptosis, and inhibiting [Ca^2+^]_i_ release 11-oxo-Mogrol: -Reversed MK801-induced phosphorylation inactivation of AKT and mTOR	(Ju et al. 2020)
	11-Oxo-mogrol		
**Anthraquinones**	Emodin	Inhibition of epidermal growth factor (EGF)-stimulated phosphorylation process of both ErbB1 and ErbB2 Reduction of pre-pulse inhibition and improvement of startle reponses in rats	(Mitra et al. 2022)
**Polyphenols**	Resveratrol	Each improved negative symptoms when taken as an adjunctive therapy in a double-blind, randomized, placebo-controlled study Curcumin-loaded nanophytosome alleviated Ketamine-induced brain injury, showing a marked reduction in the depressive and anxiety-like behaviors, memory deficits, and oxidative stress markers in cortical and subcortical tissues	(Miodownik et al. 2019; Samaei et al. 2020; Moghaddam et al. 2021)
	Curcumin		
	Epigallatocathechin-3-gallate	Neuroprotective activity attributed to scavenging free radicals, reducing oxidative stress and efficacy against neuroinflammatory processes	(Li et al. 2024b)
**Flavonoids**			
Anthocyanins	Petunidin chloride	They inhibited pentosidine synthesis in human plasma (*In-vitro* synthesis assay) by > 80% with pentunidin chloride showing the best inhibitory activity, with IC_50_ of 0.220 mM; 85-fold higher than that of pyridoxamine. Oxygen Radical Antioxidant Capacity (ORAC) values demonstrated correlation between their inhibitory effects and antioxidant activity SAR: Compounds with flavonoid basic nucleus showed the best activity results among the tested compounds, and the higher the number of hydroxyl moieties the stronger the activity	(Asakura et al. 2024)
	Delphinidin chloride		
	Cyanidin chloride		
	Delphinidin 3-galactoside chloride		
	Petunidin 3-galactoside chloride		
Chalcones	3-(3,4-Dimethoxyphenyl)−1-(4- methoxyphenyl) prop-2-en-1-one	Prominent neuroprotective action; via downregulating ATPases activity and Acetylcholine production in different brain regions of rat models	(Venkataramaiah 2020; Chintha and Wudayagiri 2022)
Flavones	Baicalin	Baicalin is a prodrug which gives the aglycone part; baicalein in GIT They both are direct inhibitors of POP enzyme in dose-dependent manner, with IC_50_ of 12 ± 3 μM for baicalin, and 36 ± 6 μM for baicalein Since both compounds gave rather similar POP inhibitory activity, it is concluded that the sugar part does not affect the activity Baicalein crosses barriers of gastrointestinal tract (GIT) and blood brain barrier (BBB), the sugar prevents passive diffusion of baicalin through the BBB	(Tarragó et al. 2008)
	Baicalein		
Flavonols	Quercetin	Flavonols such as Quercetin displayed inhibitory effects on poly (ADP-ribose) polymerase (PARP) enzyme; a transcription factor which regulates neuroinflammation by stimulating NF-kB Negative allosteric GABA_A_ receptor modulator Usage as adjuvant therapy improved both positive and negative symptoms in a clinical study and improved the Brief Psychiatric Rating Scale BPRS score	(Schwartz 2016; Fan et al. 2018; Matrisciano 2023)
	Rutin	“U-shaped” dose-dependent inhibiting effect on climbing and stereotyped behaviors, ameliorating positive symptoms Antidopaminergic activity	(Pandy and Vijeepallam 2017; Calabrese et al. 2024)
	Fisetin	Stimulation of BDNF expression Maintaining hippocampal synaptic plasticity and memory functions by means of increasing the surface expression of the GluA1 subunit of AMPA receptor as well as maintaining the phosphorylation of the AMPA receptors, as well as CaMKII, CREB, ERK1/2	(Hassan et al. 2022)
	Icariin	Anti-schizophrenic activity on rat model and molecular pharmacology, where it improves the spatial learning and memory abilities via TNF signaling pathway	(Liu et al. 2024)
Flavonolignans	Silymarins	Prevention of KET-induced Schizophrenia-like behavior in mice model, through normalization of the neurotrophic and neurochemical changes and inhibition of neuroinflammation	(Ben-Azu et al. 2024)
**Coumarins**	Scopoletin	“U-shaped” dose-dependent inhibiting effect on climbing and stereotyped behaviors, ameliorating positive symptoms Antidopaminergic activity	(Pandy and Vijeepallam 2017)
	Urolithin A	Prevention of MK-801-induced cognitive deficits in female rats mainly through inhibitory activity against neuroinflammation and microglial activation by modulation of the NLRP3 signaling pathway	(Huang et al. 2025)
**Amino acids**	N-Acetylcysteine	Improvement in negative symptoms on total SANS and CGI scores at 24 weeks when taken as adjunctive therapy	(Tharoor et al. 2018)
**Carotenoids**	Crocin	Upregulation of silent information regulator-1 (SIRT1) and brain derived neurotrophic factor (BDNF) expression and relieving oxidative stress (in the hippocampus of neonatal rat model) Safety was confirmed in patients having SCZ at a dose of 15 mg twice daily	(Mousavi et al. 2015; Sun et al. 2020)
**Isothiocyanates**	Glucoraphanin	Supplementation during pregnancy and lactation may prevent development of neuropsychiatric disorders in offspring	(Fujita et al. 2020)
	Sulforaphane		
**Cannabinoids**	Cannabidiol (CBD)	Chronic treatment with 30 and 60 mg/kg doses reversed PPI impairment and reduced all molecular changes observed after chronic administration of an NMDAR antagonist It alleviates both positive and negative symptoms It has a pharmacological profile similar to that of atypical anti-psychotic drugs and is a safe, well-tolerated promising drug alternative for SCN	(Deiana 2013; Gomes et al. 2014)
**Polyunsaturated fatty acids**	Omega-3 fatty acids	Enhancement in BDNF, decrease in CRP, IL-6, TNF*α*, hence improving cognitive function Anti-inflammatory effects via regulating triglyceride (TG) metabolism and reduction of TNF*α* levels	(Xu et al. 2019; Tang et al. 2020)
**Vitamins**	Retinol (Vitamin A)	Low maternal vitamin A levels in the second trimester was associated with more than threefold increase in schizophrenia spectrum illnesses among adult offspring	(Bao et al. 2012)
	Nicotinamide (Vitamin B_3_)	Lower levels of them were associated significantly with SCN in a population-based case–control study Pyridoxamine inhibits accumulation of advanced glycation end products	(Cao et al. 2018; Ueno et al. 2024)
	Pyridoxine (Vitamin B_6_)		
	Vitamin B_12_	Lower plasma levels in patients vs controls	(Kale et al. 2010)
	Folic acid	Serum folate concentrations showed significant correlation with the severity of negative symptoms Lower plasma levels in patients vs control group	(Goff et al. 2004; Kale et al. 2010)
	Cholecalciferol (Vitamin D_3_)	Beneficial effects on PANSS scores, and metabolic profiles by increasing total antioxidant capacity, and decreasing hsCRP levels	(Ghaderi et al. 2019a)
	ʟ-Ascorbic acid (Vitamin C)	Reduced levels in plasma of patients vs control group Adjunctive therapy of vitamin C with atypical antipsychotic reverses ascorbic acid reduced levels in schizophrenic patients, reduces oxidative stress, and improves BPRS score	(D'Souza and D'Souza 2003; Dakhale et al. 2005)
	*α*-Tocopherol (Vitamin E)	Reduced levels in plasma of patients vs control group	(D'Souza and D'Souza 2003)

This table summarizes a wide spectrum of natural compounds classified by chemical groups and their potential therapeutic roles in the treatment or modulation of schizophrenia-related pathophysiology (SCN). Each entry includes the specific compound, its reported physiological or neuropharmacological effects, and supporting references. The mechanisms span diverse neurotransmitter systems (dopaminergic, serotonergic, glutamatergic, cholinergic), anti-inflammatory and antioxidant pathways, neurotrophic support, enzyme inhibition (e.g., prolyl oligopeptidase, acetylcholinesterase), and epigenetic modulation. These compounds have been studied across preclinical (in vitro and animal models) and clinical settings, providing a promising foundation for integrative and adjunctive therapeutic strategies in schizophrenia treatment. Structural Activity Relationship (SAR) insights, blood–brain barrier (BBB) permeability, and pharmacokinetics were considered where applicable, highlighting compounds like baicalein, cannabidiol (CBD), and omega-3 fatty acids for their translational potential. This extensive profiling aims to guide future pharmacognosy research, drug discovery, and neuropsychiatric intervention design

## Clinical trials in schizophrenia management

KarXT (xanomeline-trospium), created by Karuna Therapeutics and Bristol Myers Squibb, is an innovative strategy that targets muscarinic receptors instead of conventional dopamine pathways. It has shown substantial enhancements in both positive and negative symptoms of schizophrenia, accompanied by a good side effect profile. Nonetheless, a recent late-stage experiment failed to achieve its primary aim when utilized as an adjuvant treatment, signifying the necessity for more investigation (Azargoonjahromi 2024).

Evenamide, a glutamate modulator under investigation by Newron Pharmaceuticals, is in Phase II trials targeting treatment-resistant schizophrenia. Interim results have been promising, suggesting potential benefits for patients unresponsive to existing treatments (Singh et al. 2024).

NBI-1117568, a selective muscarinic M4 receptor agonist from Neurocrine Biosciences, is undergoing Phase III trials. While early results showed symptom improvement, they were less robust compared to other emerging therapies, highlighting the competitive landscape of novel antipsychotic development (Ye et al. 2025).

Prescription digital therapeutics (DTx): CT-155, a mobile-based digital therapeutic, has received FDA breakthrough device designation. It digitizes behavioral therapy to support daily management of schizophrenia symptoms (Lutz et al. 2022).

GLP-1 Receptor Agonists (e.g., Semaglutide): Originally for diabetes, these agents are being explored for mental health benefits, including potential improvements in schizophrenia symptoms, possibly due to anti-inflammatory effects (Sass et al. 2023).

## Patents in schizophrenia management

Combination therapy with α7 nicotinic acetylcholine receptor agonists and 5-HT3 receptor modulators: This patent outlines a method for treating schizophrenia using a combination that targets both α7 nicotinic acetylcholine receptors and 5-HT3 receptors, aiming to enhance therapeutic efficacy (Granger and Barnett 2022).

Flavonoid-based treatments: A patent describes the use of flavonoids, such as quercetin and luteolin, administered orally to alleviate symptoms of psychosis, including schizophrenia (Melrose 2023).

Beta-caryophyllene (BCP) compositions: This patent discusses the use of BCP, a CB2 receptor agonist, in treating schizophrenia, highlighting its potential anti-inflammatory and neuroprotective properties (Ricardi et al. 2024).

These developments underscore a shift towards targeting alternative neural pathways and employing combination therapies to improve outcomes for individuals with schizophrenia. Ongoing research and clinical trials continue to expand the therapeutic landscape, offering hope for more effective and personalized treatment options.

## Future directions in schizophrenia management

Despite advancements in pharmacological treatments, cognitive dysfunction, treatment resistance, and metabolic side effects remain significant challenges in schizophrenia management. Current pharmacological treatments primarily target dopaminergic and serotonergic pathways, but they often fail to address the full spectrum of symptoms, including cognitive impairment and negative symptoms. Future research should focus on integrative therapeutic strategies that combine pharmacological agents with natural compounds to improve efficacy and safety while minimizing side effects. Additionally, advances in personalized medicine and the increasing understanding of the gut-brain axis open new avenues for innovative treatment approaches (Hoenders et al. 2018; Beger et al. 2020; Sethi and Ford 2022; Ju et al. 2023; Mosquera et al. 2024).

## Personalized and precision medicine approaches

- Biomarker-guided treatments**:** The identification of oxidative stress, inflammatory, and genetic markers can facilitate tailored interventions using natural compounds. Advanced neuroimaging and metabolomic analyses can provide a deeper understanding of individual patient profiles, allowing for more precise interventions (Korczowska-Łącka et al. 2023; Krzyściak et al. 2024; Vo and Trinh 2024).
- Pharmacogenomics**:** Investigating genetic polymorphisms that influence drug response can optimize combination therapies, improving treatment outcomes and minimizing side effects. Understanding how genetic variations impact metabolism and receptor activity can help refine medication regimens, leading to better adherence and fewer adverse reactions (Micaglio et al. 2021; Pirmohamed 2023; Anghel et al. 2024).
- Epigenetic modulation**:** Research into how environmental factors influence gene expression may lead to novel interventions that modify the epigenetic landscape, potentially reversing disease progression or enhancing treatment efficacy (Lisoway et al. 2021; Farrokhi et al. 2023).

## Natural compound-based drug development

- Nanoformulations for enhanced bioavailability**:** Many bioactive compounds, such as curcumin, resveratrol, and flavonoids, exhibit poor bioavailability. Nanoparticle-based delivery systems can enhance their absorption and therapeutic efficacy. Encapsulation methods, such as liposomal delivery and polymeric nanoparticles, offer promising solutions for improving the pharmacokinetics of these compounds (Yang et al. 2020; Sharifi-Rad et al. 2021; Hassanizadeh et al. 2023).
- Synergistic combinations with standard therapies: Studying interactions between natural compounds and antipsychotic medications can help reduce drug dosages, thereby minimizing side effects while maintaining efficacy. Certain flavonoids and polyphenols have shown the ability to modulate neurotransmitter systems and neuroinflammation, potentially complementing existing pharmacological treatments (Le et al. 2022; Bellavite 2023; Duda-Chodak and Tarko 2023).

## Gut-brain axis and microbiome-based therapies

- Probiotics and polyphenols**:** Modulating gut microbiota through probiotics and polyphenol-rich diets may positively influence neurotransmission and reduce inflammation, contributing to improved mental health. Recent studies suggest that dysbiosis, or an imbalance in gut microbiota, may contribute to psychiatric disorders, including schizophrenia (İnce Palamutoglu et al. 2024; Munawar et al. 2024; Vasileva et al. 2024).
- Dietary interventions: Exploring ketogenic and polyphenol-rich diets can enhance the response to antipsychotic treatments by influencing metabolic and neuroinflammatory pathways. A ketogenic diet, for example, has been shown to improve mitochondrial function and reduce oxidative stress, both of which are implicated in schizophrenia (Grabska-Kobyłecka et al. 2023; Rog et al. 2024).
- Microbiome-derived metabolites: Short-chain fatty acids (SCFAs) produced by gut bacteria may play a critical role in modulating neuroinflammation and neurotransmitter synthesis. Investigating how dietary changes affect SCFA production could provide new therapeutic opportunities (Moțățăianu et al. 2023; Qu et al. 2024).

## Conclusions

Over a thousand phytochemicals have been discovered too far, and they can be taken from a variety of sources, including whole grains, fruits, vegetables, nuts and herbs. Schizophrenia is a condition characterized by changes in brain structures such as loss of grey matter, expanded ventricles, and reduction of dendritic spines from pyramidal neurons in the cortex, which can manifest as delusions, hallucinations, extremely disordered thinking, disorganized behavior, flat affect, amotivation, energy, and failure to maintain hygiene, among many other symptoms. Natural medications, such as phytochemicals, have shown therapeutic potential in the treatment of schizophrenia by modulating oxidative stress, neuro-inflammation, immune system changes, and downstream signaling pathways that are hallmarks of the disease. Schizophrenia is a multifaceted disease with a complicated etiology and pathophysiology that requires numerous targeted therapy options to improve both positive and negative symptoms, as well as cognitive impairment. Alkaloids, glycosides, terpenes, terpenoids, polyphenols, flavonoids, poly-propanoids, steroidal lactones, and amino acids are among the primary types of phytochemicals that have demonstrated anti-schizophrenic efficacy in preclinical studies. Apomorphine, luteolin, apigenin, caryophyllene, cannabinoids, baicalin, and reserpine are some of the phytochemicals that have shown anti-schizophrenic activity in human research. As a result, it is feasible to speculate that phytochemicals could be potential candidates for generating novel medicines with preventive and therapeutic advantages against schizophrenia. Furthermore, further preclinical and clinical research is needed to establish pharmacokinetic and toxicity studies of phytochemicals, as well as the best potential combinations to reduce unwanted side effects. Unfortunately, despite the phytochemicals'great neuroprotective potential against schizophrenia, no long-term studies of these medicines against schizophrenia have been conducted to investigate their effects on disease progression. Furthermore, specific doses and combinations of phytochemicals should be studied in clinical trials to demonstrate efficacy and safety in schizophrenia patients.

## Data Availability

All source data for this work (or generated in this study) are available upon reasonable request.
